# Anti-plasmodial activity of Norcaesalpin D and extracts of four medicinal plants used traditionally for treatment of malaria

**DOI:** 10.1186/s12906-017-1673-8

**Published:** 2017-03-24

**Authors:** Ramadhani Selemani Omari Nondo, Mainen Julius Moshi, Paul Erasto, Pax Jessey Masimba, Francis Machumi, Abdul Waziri Kidukuli, Matthias Heydenreich, Denis Zofou

**Affiliations:** 1 Department of Biological and Pre-Clinical Studies, Institute of Traditional Medicine, Muhimbili University of Health and Allied Sciences, P.O.Box 65001, Dar es Salaam, Tanzania; 2 National Institute for Medical Research, P.O.Box 9653, Dar es Salaam, Tanzania; 3 Department of Natural Products Development and Formulation, Institute of Traditional Medicine, Muhimbili University of Health and Allied Sciences, P.O.Box 65001, Dar es Salaam, Tanzania; 4 Institut für Chemie, Universität Potsdam, Karl-Liebknecht, Str. 24-25, 14476, Golm, Germany; 5 Biotechnology Unit, University of Buea, P.O.Box 63, Buea, South West Region, Cameroon

**Keywords:** Antiplasmodial, norcaesalpin D, *E. schliebenii*, *H. pubescens*, *D. melleri*, *C. bonducella*

## Abstract

**Background:**

Malaria is an old life-threatening parasitic disease that is still affecting many people, mainly children living in sub-Saharan Africa. Availability of effective antimalarial drugs played a significant role in the treatment and control of malaria. However, recent information on the emergence of *P. falciparum* parasites resistant to one of the artemisinin-based combination therapies suggests the need for discovery of new drug molecules. Therefore, this study aimed to evaluate the antiplasmodial activity of extracts, fractions and isolated compound from medicinal plants traditionally used in the treatment of malaria in Tanzania.

**Methods:**

Dry powdered plant materials were extracted by cold macerations using different solvents. Norcaesalpin D was isolated by column chromatography from dichloromethane root extract of *Caesalpinia bonducella* and its structure was assigned based on the spectral data. Crude extracts, fractions and isolated compound were evaluated for antiplasmodial activity against chloroquine-sensitive *P. falciparum* (3D7), chloroquine-resistant *P. falciparum* (Dd2, K1) and artemisinin-resistant *P. falciparum* (IPC 5202 Battambang, IPC 4912 Mondolkiri) strains using the parasite lactate dehydrogenase assay.

**Results:**

The results indicated that extracts of *Erythrina schliebenii, Holarrhena pubescens, Dissotis melleri* and *C. bonducella* exhibited antiplasmodial activity against Dd2 parasites. Ethanolic root extract of *E. schliebenii* had an IC_50_ of 1.87 μg/mL while methanolic and ethanolic root extracts of *H. pubescens* exhibited an IC_50_ = 2.05 μg/mL and IC_50_ = 2.43 μg/mL, respectively. Fractions from *H. pubescens* and *C. bonducella* roots were found to be highly active against K1, Dd2 and artemisinin-resistant parasites. Norcaesalpin D from *C. bonducella* root extract was active with IC_50_ of 0.98, 1.85 and 2.13 μg/mL against 3D7, Dd2 and IPC 4912-Mondolkiri parasites, respectively.

**Conclusions:**

Antiplasmodial activity of norcaesalpin D and extracts of *E. schliebenii, H. pubescens, D. melleri* and *C. bonducella* reported in this study requires further attention for the discovery of antimalarial lead compounds for future drug development.

## Background

Malaria is an old mosquito borne disease which is still a public health concern particularly in Africa [1]. Globally, about 214 million people were recorded to suffer from malaria in 2015 of which majority are found in sub-Saharan Africa [2].

Efforts to control malaria have mainly depended on the availability of effective antimalarial drugs and insecticides to reduce both infections and transmission [3]. Use of insecticide-treated bed nets (INTs), indoor residual spraying (IRs), chemoprophylaxis in pregnant mothers and increased access to effective antimalarial drugs have significantly reduced morbidity and mortality, especially, in malaria endemic areas [4]. However, these achievements are undermined by the existence of malaria parasites and mosquitoes which are resistant to antimalarial drugs and pesticides, respectively [5].

In the early 2000s *P. falciparum* parasites resistant to mefloquine, chloroquine, quinine, proguanil, atovaquone and sulphadoxine-pyrimethamine, but not, artemisinins were reported [6]. The WHO responded by restricting artemisinin monotherapies and recommended use of artemisinin-based combination therapies, ACTs [7] in which the artemisinins are combined with long acting drugs. Subsequently, increased access to the ACTs has contributed significantly to reducing both infections and malaria-related deaths, especially in sub-Saharan Africa where the transmission is high [8]. Despite this positive outcome, a recent report from South East Asia revealed that *P. falciparum* parasites resistant to artemisinin have now been identified in Myanmar, Cambodia, Thailand, Viet Nam and Lao People’s Democratic Republic [9]. Recently clinical failure to dihydroartemisinin-piperaquine combination therapy was reported [10], suggesting that the problem is increasing and thus there is need for development of new drugs. To address this challenge, researches which will identify new chemical compounds with antimalarial properties are continuously needed. Studying medicinal plants used in traditional medicine for the treatment of malaria is among the many approaches used to identify new compounds with antimalarial properties [11]. Therefore, this paper reports the antiplasmodial activity of extracts, fractions and one compound from medicinal plants traditionally used for the treatment of malaria in Tanzania.

## Methods

### Malaria parasites

Chloroquine-sensitive *P. falciparum* (3D7), chloroquine-resistant *P. falciparum* (Dd2 and K1) and artemisinin-resistant *P. falciparum* (IPC-5202 Battambang-Cambodia 2011 and IPC-4912 Mondolkiri-Cambodia 2011) strains were used. All parasites were obtained from BEI-resources (MR4/ATCC^®^ Manassas, VA, USA).

### Chemicals and reagents

Silica gel (230–400 mesh, 60 Å; Sigma, Steinheim, Germany), dichloromethane (Carlo erba^®^, Val-de-Reuil, France), ethyl acetate (Carlo erba^®^), ethanol (Carlo erba^®^), methanol (Carlo erba^®^), dimethyl sulfoxide (Carlo erba^®^), thin layer chromatography (TLC) plates (60 F_254_, Merck, Darmstadt, Germany), Albumax II (GIBCO^™^, Invitrogen, USA), RPMI-1640 (Sigma^®^), Foetal Bovine Serum (FBS, BioWhittaker^®^, Verviers, Belgium), were used. All chemicals and other materials used in this study were purchased through local suppliers in Tanzania and in Cameroon, and directly from SIGMA (Sigma^®^, Steinheim, Germany).

### Collection and extraction of plant materials

Previous studies indicate that decoctions of *E. schliebenii* roots and stem bark*, H. pubescens* roots*, C. bonducella* roots and leaves, and decoction of *D. melleri* aerial parts are used in traditional medicine for the treatment of malaria [12–14]. *Dissotis melleri* Hook.f (Melastomataceae, voucher No. RN 55) aerial parts were collected from Buzi Kishura village in Kagera region and *Caesalpinia bonducella* (L.) Flem (Caesalpiniacea, Voucher no. RN 93) roots were collected from Gezaulole village in Dar es Salaam region, Tanzania. *Holarrhena pubescens* Huch-Ham (Apocynaceae, voucher No. 4665) roots and *Erythrina schliebenii* Harms (Fabaceae, voucher No. 4661) stem bark and roots were collected from Mchakama village in Lindi region, Tanzania. The plants were identified by a botanist, Mr. Selemani Haji, and the voucher specimens are deposited in the Herbarium of the Institute of Traditional Medicine, Muhimbili University of Health and Allied Sciences.

Dry powdered plant materials were extracted by cold maceration using 80% ethanol, methanol, dichloromethane, or water. The crude extracts were dried in vacuo at 50 °C. Aqueous extracts were dried by freeze-drying and the dry extracts were kept at −20 °C.

### Fractionation of root extract of *H. pubescens*

Methanolic root extract of *H. pubescens* and dichloromethane root extract of *C. bonducella* were selected for fractionation after showing both in vitro antiplasmodial activity reported in this study and in vivo antimalarial activity reported in our previous study [15]. The methanolic root extract of *H. pubescens* was fractionated by column chromatography with silica gel as the stationary phase. The column was eluted by solvents of increasing polarity starting with petroleum ether (100%). The polarity of eluting solvent was increased using a step gradient of ethyl acetate in petroleum ether in the following ratios: ethyl acetate/petroleum ether (1:9), ethyl acetate/petroleum ether (1:4), ethyl acetate/petroleum ether (2:3); ethyl acetate/petroleum ether (1:1), ethyl acetate/petroleum ether (7:3); ethyl acetate/petroleum ether (4:1), ethyl acetate (100%) and finally eluted with methanol (100%). After analysis by thin layer chromatography (TLC), similar fractions were combined to obtain 11 major fractions coded as methanolic root extract of *H. pubescens* (HPRM-1 to 11). All fractions were air-dried and evaluated for antiplasmodial activity against chloroquine-sensitive *P. falciparum* (3D7), chloroquine-resistant *P. falciparum* (Dd2 and K1) and artemisinin-resistant *P. falciparum* (IPC-5202 and IPC-4912) parasites.

### *Isolation and characterization of pure compound from C. bonducella root* extract

#### General experimental procedure

Isolation of the compounds was done by column chromatography with silica gel as the stationary phase and further purified by size exclusion column chromatography using Sephadex^®^ LH-20. Separation was monitored by TLC in aluminium plates pre-coated with silica gel and spots were visualized under UV light (254 nm) and after spraying with vanillin plus heat [16]. ^1^H, ^13^C, COSY, HSQC, HMBC and NOESY spectral data were recorded on a Bruker Avance (600 MHz) NMR spectrometer in CD_2_Cl_2_ solvent with the residual solvent signal (^1^H: 5.31, ^13^C: 53.7 ppm) as internal standard. EI-MS spectrum was recorded on GC-MS TRACE DSQII single quadruple mass spectrometer.

#### Extraction, fractionation, isolation and characterization of compound

The dry-powdered roots of *C. bonducella* (1.7 Kg) were macerated twice in dichloromethane for 24 h at room temperature. The extract was dried *in vacuo* to give 46.3 g, out of which 40 g were fractionated in silica gel column chromatography and eluted by solvents of increasing polarity starting with 2.5 L of 100% petroleum ether (yield 1.7 g) followed by 2.0 L of 1:1 petroleum ether/dichloromethane (yield 6.5 g), 2.5 L of 100% dichloromethane (yield 12.0 g), 2.5 L of 1:1 dichlromethane/ethyl acetate (yield 16.7 g) and 0.8 L of 100% ethyl acetate (yield 1.0 g). All fractions were screened for in vivo antimalarial activity against *P. berghei* ANKA parasites at a dose of 200 mg/kg/day for a total of four daily doses given orally. Among the tested fractions, 1:1 dichloromethane/ethyl acetate fraction showed highest in vivo activity and therefore was selected for further fractionation and isolation of active compounds. About 16.0 g of 1:1 dichloromethane/ethyl acetate fraction was chromatographed in a silica gel column and eluted by solvents of increasing polarity starting with 1:1 petroleum ether/dichloromethane, then 1:4 petroleum ether/dichloromethane, 100% dichloromethane and 9:1 dichloromethane/ethyl acetate. Fraction number 12 obtained from 9:1 dichloromethane/ethyl acetate showed some crystals and this fraction was further purified by sephadex column eluted by 100% ethyl acetate to produce 12 sub-fractions in which sub-fraction number 3 was found to be white amorphous solid pure compound.

### In vitro antiplasmodial activity

Chloroquine-sensitive *P. falciparum* (3D7), chloroquine-resistant *P. falciparum* (Dd2 and K1) and artemisinin-resistant *P. falciparum* (IPC 5202 Battambang-Cambodia 2011 and IPC 4912 Mondolkiri- Cambodia 2011) strains were cultured in vitro according to the method of Trager and Jensen [17]. Parasites were grown in uninfected O^+^ human red blood cells as host cells and maintained in RPMI-1640 culture medium supplemented with NaHCO_3_ (2 mg/mL), hypoxanthine (10 μg/mL), glucose (2 mg/mL), albumax II (1%) and gentamicin (10 μg/mL). The parasite cultures were incubated at 37 °C in 5% CO_2_, 5% O_2_ and 90% N_2_. All solutions were filter-sterilized by 0.22 μm syringe-adapted filters (Corning^®^, NY, USA).

In vitro antimalarial activity of extracts, fractions and norcaesalpin D isolated from *C. bonducella* roots was assessed by parasite lactate dehydrogenase (pLDH) assay [18]. Non-synchronized 1% parasitized red blood cells (pRBCs, mostly rings and trophozoites) at 2% haematocrit (hct) in 96 well microtiter plates (Costar^®^, Corning, NY, USA) were incubated with different concentrations of extract, fractions or norcaesalpin D. Quinine was used as a standard drug. Each concentration was tested in triplicate and each experiment was done twice. Wells with only 1% pRBCs at 2% hct without extract, fraction or drug were included as negative controls (100% parasite growth) while wells with uninfected red blood cells only at 2% hct served as blank controls. Parasite cultures with extracts, fractions or compound were maintained for 48 h at 37 °C in 5% CO_2_, 5% O_2_, and 90% N_2_. After 48 h of incubation, the plates were frozen overnight at −20 °C and antimalarial activity was determined by pLDH assay performed as described previously [19]. Concentrations inhibiting 50 % of the parasite growth (IC_50_) were determined by HN-NonLin V1.1, 2002 software (http://www.meduniwien.ac.at/user/harald.noedl/malaria/software.html) (Noedl H, 2002. Armed Forces Research Institute for Medical Sciences, Bangkok, Thailand).

### Cytotoxicity

Cytotoxicity was evaluated on LLC-MK2 monkey kidney epithelial cells. Cells were grown in culture medium prepared from solid RPMI-1640 with L-glutamine and 25 mM HEPES (Sigma^®^, Steinheim, Germany). The medium was supplemented with NaHCO_3_ (2 mg/mL), hypoxanthine (10 μg/mL), glucose (11.1 mM), 10% FBS (BioWhittaker^®^, Verviers, Belgium) and gentamicin (5 μg/mL). Adherent cells detached by trypsin-EDTA treatment were distributed into 96 well microtiter plates at 10,000 cells in 100 μL per well. The cells were incubated for 48 h to allow them to attach before adding the extracts. After 48 h, the medium was removed completely from each well, and 100 μL of fresh culture medium was then added. Thereafter, 100 μL of crude extracts (400 μg/mL) were added in row H and then serially diluted two fold to give concentrations ranging from 200 to 3.125 μg/mL. Cells in row A served as controls without drug (100% growth). The cells with or without extracts/fractions/compound were incubated at 37 °C in atmosphere of 5% CO_2_ air for 72 h before determining their viability. Each concentration was tested in triplicate and each experiment was done twice. Cell viability was determined by MTT assay and cytotoxic activity was determined according to a method used in our previous study [20]. Gleevec^®^ (Imatinib) was used as a cytotoxic drug. Selectivity index (SI) corresponding to the ratio between cytotoxic activity and antiplasmodial activity was calculated as follows: SI (Plasmodium) = CC_50_ (LLC-MK2)/ IC_50_ (*P. falciparum*).

## Results

### Structure elucidation of Norcaesalpin D

**Fig. 1:**
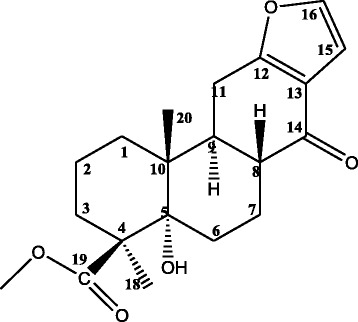
Structure of Norcaesalpin D (methyl-12,16-epoxy-5α-hydroxynorcasa-12,15-dien-14-one-19-carboxylate)

Norcaesalpin D (Fig. 1) was isolated as a white amorphous solid pure compound from the dichloromethane root extract of *C. bonducella*. The EI-MS gave molecular ion peak [M^+^] at *m/z* 346 consistent with the molecular formula C_20_H_26_O_5_. The ^1^H–NMR revealed twenty five protons including methyl doublet at δ 0.87, two methyl singlets at δ 1.15 and at δ 3.65 (methoxy protons) together with two olefinic protons of a 2,3-disubstituted furan ring at δ 6.59 (dd, J = 2.0, 0.4) and at δ 7.32 (d, J = 1.8). The ^13^C–NMR showed signals for twenty carbons of which two were carbonyl carbons at δ 195.9 corresponding to C-14 ketone group and at δ 177.2 representing C-19 carbonyl ester. One signal for methoxy carbon at δ 51.7 was observed. In addition to that, four signals for olefinic carbons at δ 167.1 (C-12), δ 119.8 (C-13), δ 106.4 (C-15) and δ 143.1 (C-16) of furan ring attached to another ring were observed. The significantly downfield chemical shift value of C-12 was attributed to resonance of a conjugated carbonyl residue at C-14. Its structure (Fig. 1) was assigned (Table 1) based on ^1^H, ^13^C, COSY, HSQC, HMBC and NOESY spectral data. The compound was identified as norcaesalpin D, previously isolated from the dichloromethane root extract of *C. bonducella* and its structure was confirmed by comparing with previously reported data [21]; however no antiplasmodial activity was reported before.

**Table 1:** ^1^H–NMR and ^13^C–NMR (δ values; *J* values in Hz) data for Norcaesalpin D

Position	Observed value (600 MHz in CD_2_Cl_2_)		Reported value by Roach et al., 2007 (500 MHz in CDCl_3_)	
	δ_C_ mult	δ_H_, mult (*J* in Hz)	δ_C_	δ_H_, mult (*J* in Hz)
1	32.0	1.59, m 1.39, m	31.6	1.40, m 1.58, m
2	18.9	1.59, m 1.92, m	18.7	1.53, m 1.86, m
3	31.9	1.56, m 1.90, m	31.7	1.64 1.94
4	49.2		49.0	
5	76.6		76.4	
6	28.2	1.84, ddd (14.9, 4.2, 2.6) 2.37, td (14.6, 4.8)	27.9	1.88, m 2.40, m
7	21.8	1.55, m 2.24, m	21.5	1.60, m 2.33, m
8	44.3	2.31,td (11.9, 4.8)	44.2	2.33, m
9	44.7	2.59, m	44.5	2.62, m
10	41.9		41.7	
11	23.4	2.61, m 2.80, m	23.2	2.81, m
12	167.1		166.8	
13	119.8		119.8	
14	195.9		196.1	
15	106.4	6.59, dd (2.0, 0.4)	106.5	6.64, d (2.0)
16	143.1	7.32, d (1.8)	142.2	7.30, d (2.0)
17	-		-	
18	23.7	1.15, s	23.7	1.19, s
19	177.2		177.2	
20	15.1	0.87, d (0.6)	15.1	0.89, s
19-OMe	51.7	3.65, s	51.7	3.69, s

Assignments were based on COSY, HSQC and HMBC experiments

### In vitro antiplasmodial and cytotoxic activity of norcaesalpin D and extracts of four plants

Antiplasmodial activity of extracts of *E. schliebenii, H. pubescens, D. melleri* and *C. bonducella* was determined against chloroquine-resistant *P. falciparum* Dd2 strains. The results showed that all extracts were active with IC_50_ < 25 μg/mL. Among the extracts investigated, root extracts of *E. schliebenii* and *H. pubescens* possessed the highest antiplasmodial activity with IC_50_ < 5 μg/mL. The ethanolic root extract of *E. schliebenii* had an IC_50_ of 1.87 μg/mL while methanolic and ethanolic root extracts of *H. pubescens* exhibited an IC_50_ = 2.05 μg/mL and IC_50_ = 2.43 μg/mL, respectively. The aqueous extract of *E. schliebenii* stem bark (IC_50_ = 7.04 μg/mL) and ethanolic extract of *D. melleri* (IC_50_ = 8.23 μg/mL) demonstrated promising antiplasmodial activity while dichloromethane root extract of *C. bonducella* exhibited moderate antiplasmodial activity with IC_50_ of 24.05 μg/mL (Table 2). In addition, results of cytotoxicity study revealed that all extracts were non-toxic to mammalian (LLC-MK2) cells with CC_50_ > 30 μg/mL cut-off point (Table 2).

**Table 2:** In vitro anti-plasmodial activity and cytotoxicity of plant extracts

Plant (Family)	Part (extract)	Antiplasmodial activity	Cytotoxicity	Selectivity index (SI)
		IC_50_ on Dd2 (μg/mL ± SD)	CC_50_ on LLC-MK2 (μg/mL ± SD)	
*Erythrina schliebenii* (Fabaceae)	R (80% Ethanol)	1.87 ± 0.44	>200	>109
	SB (H_2_O)	7.04 ± 0.72	>200	>28.4
*Holarrhena pubescens* (Apocynaceae)	R (80% Ethanol)	2.43 ± 0.15	>200	>82.3
	R (Methanol)	2.05 ± 0.56	>200	>97.6
*Dissotis melleri* Hook.f (Melastomataceae)	AP (80% Ethanol)	8.23 ± 1.32	83.33 ± 3.31	10.1
*Caesalpinia bonducella* (Caesalpinaceae)	R (Dichloromethane)	24.05	>200	>8.3
QN		0.06 ± 0.01		
Gleevec^®^ (Imatinib)			19.43 ± 2.87	

*R* root, *SB* stem bark, *AP* aerial parts (stem + leaves), *QN* quinine

Two fractions from the methanolic root extract of *H. pubescens* coded as HPRM-5 and 7 were evaluated for antiplasmodial activity against chloroquine-sensitive *P. falciparum* (3D7), chloroquine-resistant (K1) and artemisinin-resistant strains (IPC 5202 Battambang and IPC 4912 Mondolkiri). Although the results revealed both fractions were active, HPRM-7 had high activity against all *P. falciparum* strains tested. Fraction HPRM-5 obtained from 40% ethyl acetate/petroleum ether column fractions inhibited the growth of *P. falciparum* 3D7 (IC_50_ = 3.14 μg/mL), K1 (IC_50_ = 5.23 μg/mL), IPC 5202 Battambang (IC_50_ = 11.46 μg/mL) and IPC 4912 Mondolkiri (IC_50_ = 8.61 μg/mL). Similarly, fraction HPRM-7 from 70% ethyl acetate/petroleum ether column fractions exhibited strong antiplasmodial activity against 3D7 with IC_50_ = 1.2 μg/mL, K1 (IC_50_ = 2.18 μg/mL), and IPC 5202 Battambang IC_50_ = 2.52 μg/mL) and promising activity against IPC 4912 Mondolkiri with IC_50_ = 5.95 μg/mL (Table 3).

**Table 3:** In vitro antiplasmodial activity of fractions from *H. pubescens* methanolic root extract

Fraction	Antiplasmodial activity IC_50_ ± SD (μg/mL)				Cytotoxicity CC_50_ ± SD (μg/mL) LLC-MK2 cells
	3D7	K1	IPC 5202 Battambang- Cambodia 2011	IPC 4912 Mondolkiri- Cambodia 2011	
Fraction HPRM- 5	3.14 ± 0.18	5.23 ± 0.03	11.46 ± 0.2	8.61 ± 0.97	>200
Fraction HPRM- 7	1.20 ± 0.35	2.18 ± 0.95	2.52 ± 1.8	5.95 ± 2.79	>200
QN	0.052 ± 0.01	0.060 ± 0.01	0.074 ± 0.0	0.068 ± 0.005	>200

*HPRM-5* 40% ethyl acetate/petroleum ether column fraction of *H. pubescens* methanolic root extract, *HPRM-7* 70% ethyl acetate/petroleum ether column fraction of *H. pubescens* methanolic root extract, *QN* quinine

The 1:1 dichloromethane/ethyl acetate column fraction and its sub-fraction (sub-fraction 1) from dichloromethane crude extract of *C. bonducella* root exhibited high to moderate antiplasmodial activity against *P. falciparum* 3D7, *P. falciparum* Dd2 and *P. falciparum* IPC 4912 Mondolkiri. Generally, the activity of these fractions increased with purity of the fraction (Table 4). Norcaesalpin D isolated from sub-fraction 1 was active with IC_50_ of 0.98, 1.85 and 2.13 μg/mL against chloroquine-sensitive (3D7), chloroquine-resistant (Dd2) and artemisinin-resistant (IPC 4912 Mondolkiri) *P. falciparum* parasites, respectively. Cytotoxicity evaluation showed that norcaesalpin D is non-toxic to mammalian cells with CC_50_ > 200 μg/mL (Table 4).

**Table 4:** Anti-plasmodial activity of Norcaesalpin D and fractions from *C. bonducella* roots

Fraction/Compound	Antiplasmodial activity IC_50_ ± SD (μg/mL)			Cytotoxicity CC_50_ ± SD (μg/mL)	Selectivity index (SI) against IPC-4912
	3D7	Dd2	IPC 4912 Mondolkiri Cambodia 2011	CC_50_ on LLC-MK2 (μg/mL ± SD)	
1:1 DCM/EtoAC fraction	4.08 ± 2.87	9.33 ± 2.09	2.99 ± 4.84	76.09 ± 8.44	25.4
Sub-fraction 1 (Semi-pure)	3.06 ± 0.80	2.26 ± 0.97	5.12 ± 1.0	> 200	>39.1
Norcaesalpin D compound	0.98 ± 0.03 (2.83 μM)	1.85 ± 0.05 (5.35 μM)	2.13 ± 0.7 (6.16 μM)	> 200 (>578 μM)	>93.9
QN	0.052 ± 0.01	0.06 ± 0.01	0.068 ± 0.005		

1:1 DCM/EtoAC =1:1 dichloromethane/ethyl acetate fraction of dichloromethane root extract of *C. bonducella*; Sub-fraction 1 = column fraction of 1:1 DCM/EtoAC fraction; Norcaesalpin D isolated from sub-fraction1

## Discussion

This study aimed to assess the antiplasmodial activity of extracts of *E. schliebenii* roots and stem bark*, H. pubescens* roots*, D. melleri* aerial parts and *C. bonducella* roots. Also, the study assessed the antiplasmodial activity of fractions and norcaesalpin D isolated from *C. bonducella* roots.

The results indicate that extracts, fractions and norcaesalpin D possess in vitro antiplasmodial activity against chloroquine-sensitive (3D7), chloroquine-resistant (Dd2 and K1) and artemisinin-resistant *P. falciparum* (IPC 5202 Battambang and IPC 4912 Mondolkiri) parasites. According to Jonville et al.*,* [22], antiplasmodial activity of extracts and fractions can be classified as high activity (IC_50_ < 5 μg/mL), promising activity (IC_50_ = 5–15 μg/mL), moderate activity (IC_50_ = 15–50 μg/mL) and inactive (IC_50_ = >50 μg/mL). Based on this, all crude extracts of *E. schliebenii, H. pubescens, D. melleri* and *C. bonducella* were active against chloroquine-resistant *P. falciparum* Dd2; however, root extracts of *E. schliebenii* and *H. pubescens* exhibited the highest antiplasmodial activity with IC_50_ < 5 μg/mL. The aqueous extract of *E. schliebenii* stem bark, ethanolic extract of *D. melleri,* and dichloromethane root extract of *C. bonducella* showed promising to moderate antiplasmodial activity with IC_50_ < 25 μg/mL (Table 2). In addition, cytotoxicity study of the extracts revealed that all extracts were non-toxic to mammalian (LLC-MK2) cells with CC_50_ > 80 μg/mL (Table 2). These findings against chloroquine-resistant *P. falciparum* parasites suggest that *E. schliebenii, H. pubescens, D. melleri* and *C. bonducella* can be potential sources for the isolation of safe and effective antimalarial lead compounds.

In our previous study we reported that extracts of *E. schliebenii* stem bark*, H. pubescens* roots and *C. bonducella* roots had in vivo antimalarial activity against *P. berghei* ANKA in mice [15]. In this study we found that extracts and fractions of these plants exhibited strong to moderate antiplasmodial activity against multi-drug resistant *P. falciparum* Dd2 malaria parasites. These findings therefore provide more scientific evidence on the antimalarial properties and the use of these plants in traditional medicines for the treatment of malaria. Furthermore, two column fractions from the active extract of *H. pubescens* roots showed high antiplasmodial activity against 3D7, K1, IPC 5202 Battambang and IPC 4912 Mondolkiri parasites. Fraction HPRM-7 from 70% ethyl acetate/petroleum ether column fractions exhibited strong antiplasmodial activity against 3D7 (IC_50_ = 1.2 μg/mL), K1 (IC_50_ = 2.18 μg/mL), and IPC 5202 Battambang IC_50_ = 2.52 μg/mL) and promising activity against IPC 4912 Mondolkiri (IC_50_ = 5.95 μg/mL) as presented in Table 3. Artemisinin-based combination therapies (ACTs) are currently the only effective drugs available for the treatment of malaria [23]. However, recent report on the emergence of *P. falciparum* parasites resistant to dihydroartemisinin-piperaquine [10] which is one of the highly effective ACTs is an indication that new molecules with antimalarial properties need to be discovered. The antiplasmodial activity observed in some of the extracts and fractions against artemisinin-resistant *P. falciparum* suggest that medicinal plants can still offer more antimalarial lead compounds for future drug development.

While the antiplasmodial properties of extracts of *E. schliebenii, H. pubescens* and *C. bonducella* were reported earlier [13, 14, 24], the antiplasmodial activity of *D. melleri* is reported for the first time by this study. Ethanolic extract of *D. melleri* aerial parts showed promising antiplasmodial activity against *P. falciparum* Dd2 parasites in vitro with IC_50_ = 8.23 μg/mL. A previous study showed that stem extracts of *Dissotis brazzae* Cogn exhibited antiplasmodial activity against *P. falciparum* chloroquine-resistant (ENT 36 strain) with IC_50_ = 6.4 μg/mL. Preliminary phytochemical analysis of the extracts of *D. brazzae* revealed the presence of alkaloids, coumarins, saponins, triterpenoids, steroids, and tannins which are associated with the antiplasmodial activity of this plant [25]. The antiplasmodial activity of *D. melleri* could therefore be an indication that the genus *Dissotis* can be a potential source for the isolation of compounds with antimalarial activity.

Dichloromethane crude extract of *C. bonducella* root was reported earlier to have dose-dependent in vivo antimalarial activity in mice [15]. The results from this study indicate that dichloromethane crude extract of *C. bonducella* root exhibited low in vitro antiplasmodial activity (Table 2). The low in vitro antiplasmodial activity observed in this study can be explained by the poor solubility of the extracts in aqueous medium. This extract dissolved completely in dimethylsulfoxide but precipitated on addition of aqueous solvents. However, dichloromethane/ethyl acetate (1:1) column fraction and its sub-fraction 1 from dichloromethane root extract of *C. bonducella* showed high in vitro antiplasmodial activity against *P. falciparum* 3D7, Dd2 and IPC 4912 Mondolkiri strains (Table 4). Norcaesalpin D (Fig. 1) is a 17-norcassane furanoditerpene isolated from the sub-fraction 1. This compound revealed strong antiplasmodial activity with IC_50_ of 0.98, 1.85 and 2.13 μg/mL against *P. falciparum* 3D7, Dd2 and IPC 4912 Mondolkiri parasites, respectively (Table 4). The antiplasmodial activity of this compound is reported for the first time by this study. A previous study indicated that this compound together with 17-O-Demethylbonducellpin C; 7-Dehydroxycaesaldekarin I; Caesaldekarin C, Caesaldekarin F, Caesaldekarin J and Caesalpin F were isolated from dichloromethane root extract of *C. bonducella* but no antiplasmodial activity was reported. Caesaldekarin C and Caesaldekarin F were reported to have cytotoxic activity against a number of cancer cell lines [21]. In addition, cassane-type diterpenoid compounds from *Caesalpinia* have been reported to have antiplasmodial properties. Norcaesalpinin E from the seeds of *C. crista* revealed high antiplasmodial activity (IC_50_ = 0.09 μM) against *P. falciparum* FCR – 3/A2 [26]. Thus, the in vitro antiplasmodial activity of norcaesalpin D observed in this study confirms the antimalarial activity of the *C. bonducella* root extract. Further evaluation of the compound for in vivo antimalarial activity is suggested.

## Conclusions

Antiplasmodial activity of the extracts of *E. schliebenii, H. pubescens, D. melleri* and *C. bonducella* reported in this study provide scientific evidence supporting their use for the treatment of malaria. Antiplasmodial activity demonstrated by norcaesalpin D against artemisinin-resistant *P. falciparum* parasites requires further attention for the discovery of antimalarial lead compounds for future use.

## Abbreviations

^13^C–NMR: Carbon-13 nuclear magnetic resonance
^1^H–NMR: Proton nuclear magnetic resonance
COSY: Correlation spectroscopy
EI-MS: Electron ionization mass spectroscopy
HMBC: Heteronuclear multiple bond correlation
HSQC: Heteronuclear single quantum correlation
MS: Mass spectroscopy
NMR: Nuclear magnetic resonance
NOESY: Nuclear overhauser effect spectroscopy
WHO: World Health Organization
